# Computationally going where experiments cannot: a dynamical assessment of dendritic ion channel currents during *in vivo*-like states

**DOI:** 10.12688/f1000research.22584.2

**Published:** 2020-06-11

**Authors:** Alexandre Guet-McCreight, Frances K. Skinner

**Affiliations:** 1 Krembil Research Institute, University Health Network, Toronto, ON, M5T 0S8, Canada; 2 Department of Physiology, University of Toronto, Toronto, ON, Canada; 3 Departments of Medicine (Neurology) and Physiology, University of Toronto, Toronto, ON, Canada

**Keywords:** Hippocampus, interneuron, computational neuroscience, ion channels, dendrites, synapses.

## Abstract

**Background: **Despite technological advances, how specific cell types are involved in brain function remains shrouded in mystery. Further, little is known about the contribution of different ion channel currents to cell excitability across different neuronal subtypes and their dendritic compartments *in vivo*. The picture that we do have is largely based on somatic recordings performed *in vitro*. Uncovering *dendritic* ion channel current contributions in neuron subtypes that represent a minority of the neuronal population is not currently a feasible task using purely experimental means.

**Methods: **We employ two morphologically-detailed multi-compartment models of a specific type of inhibitory interneuron, the oriens lacunosum moleculare (OLM) cell. The OLM cell is a well-studied cell type in CA1 hippocampus that is important in gating sensory and contextual information. We create *in vivo*-like states for these cellular models by including levels of synaptic bombardment that would occur *in vivo*. Using visualization tools and analyses we assess the ion channel current contribution profile across the different somatic and dendritic compartments of the models.

**Results: **We identify changes in dendritic excitability, ion channel current contributions and co-activation patterns between *in vitro* and *in vivo*-like states. Primarily, we find that the relative timing between ion channel currents are mostly invariant between states, but exhibit changes in magnitudes and decreased propagation across dendritic compartments. We also find enhanced dendritic hyperpolarization-activated cyclic nucleotide-gated channel (h-channel) activation during *in vivo*-like states, which suggests that dendritically located h-channels are functionally important in altering signal propagation in the behaving animal.

**Conclusions: **Overall, we have demonstrated, using computational modelling, the dynamical changes that can occur to ion channel mechanisms governing neuronal spiking. Simultaneous access to dendritic compartments during simulated *in vivo* states shows that the magnitudes of some ion channel current contributions are differentially altered during *in vivo*-like states relative to *in vitro*.

## Introduction

Since the days of Hodgkin & Huxley ^1– 4^, there have been tremendous advances in techniques to probe cellular activities ^5– 11^. However, the “gold standard” of electrophysiological experiments — patch-clamp recordings — is one of the most difficult and laborious types of experiments to perform in live animals ^10^. Although neurons have been recorded intracellularly *in vivo* ^12– 15^, inhibitory interneuron subtypes are more often recorded juxtacellularly ^16– 19^. This is because inhibitory interneurons represent a small percentage of the neuronal population, which makes it more difficult to access and patch them relative to pyramidal cells – though there are some studies in barrel cortex where interneuron patch clamp recordings have been obtained ^20, 21^. Patch-clamp experiments are vitally beneficial to our understanding since they can provide clear signals of single-cell and single-channel activity at a high temporal resolution. Using this technique in combination with ion channel blockers helps uncover the ion channel mechanisms through which cell excitability is governed. Because *in vivo* patch-clamp recordings of interneurons are so difficult to perform, not much is known about their ion channel current contribution profiles *in vivo* or how they might differ from *in vitro*. Moreover, attempting to experimentally assess different ion channel currents in *dendritic* compartments of interneuron subtypes carries with it a risk for loss of time and resources in return for only a small amount of data. Indeed, most experiments focus on attaining neuronal recordings in somata, since the comparatively thinner dendrites and axons are more difficult to patch.

Using a combination of biochemical and electrophysiological techniques, it has been shown that neurons, especially inhibitory neurons, can be characterized into many different cellular classes ^22– 27^. In the CA1 region of the hippocampus, inhibitory interneurons represent about 10–15% of neurons ^28^, and because they are more diverse than the excitatory cell population, they are also more difficult to isolate and record. As such, a lot less is known about their activities *in vivo* and the ion channel mechanisms governing their excitability, although we note that there have been several studies using calcium imaging or extracellular recording techniques to uncover interneuron firing or activation patterns during behaviour (e.g. see 16– 19, 29, 30). One such interneuron type whose firing patterns have been characterized experimentally in awake and behaving animals, is the oriens lacunosum moleculare (OLM) cell ^17, 18, 31^. In CA1, OLM cells have somata and dendrites in the stratum oriens/alveus, and receive inputs from local pyramidal cells ^32^, bistratified cells ^32^, interneuron specific 3 cells ^33^, and long-range projecting GABAergic inputs from medial septum ^33^, among others. They have axons projecting to the stratum lacunosum moleculare where they synapse onto the distal dendrites of pyramidal cells, which allows them to gate the flow of sensory information from entorhinal cortex ^32, 34^. Though OLM cell (both putative and confirmed) intrinsic properties have been studied extensively *in vitro* ^35– 41^ and computationally ^42– 47^, much remains unknown about the ion channel current profiles across their *dendritic* trees as well as how ion channel mechanisms affect OLM cell firing *in vivo* during behaviour.

Previous experimental studies have shown that neurons exhibit excitability differences *in vivo* compared to *in vitro*, due to the effects of synaptic bombardment causing “high-conductance states” ^12, 15, 48, 49^. Though these experiments have mostly focused on recordings from the excitatory cell population, there is some evidence that interneurons show similar differences *in vivo* versus *in vitro* ^50^. With morphologically-detailed cellular models containing biophysical ion channel mechanisms it is possible to create *in vivo*-like states by including a plethora of excitatory and inhibitory synaptic inputs ^12, 51, 52^. With such models, one can easily probe and record multiple ion channel current types concurrently across different dendritic compartments. In doing so, one can predict how ion channel current contributions may change between *in vitro* and *in vivo*-like states as well as across dendritic compartments.

In this work we use two morphologically-detailed models of OLM cells and bombard them with synaptic inputs so as to create *in vivo*-like states. We use these models to determine somatic and dendritic ion channel current contributions to excitability that may occur in the behaving animal. In doing so, we highlight changes in dendritic excitability, ion channel current contributions and co-activation patterns relative to *in vitro* states. Specifically, we find enhanced dendritic hyperpolarization-activated cyclic nucleotide–gated (HCN) channel activation during *in vivo*-like states, which suggests a specific role for these channels in altering signal propagation in the behaving animal.

## Methods

### Neuron models

**Figure 1.**
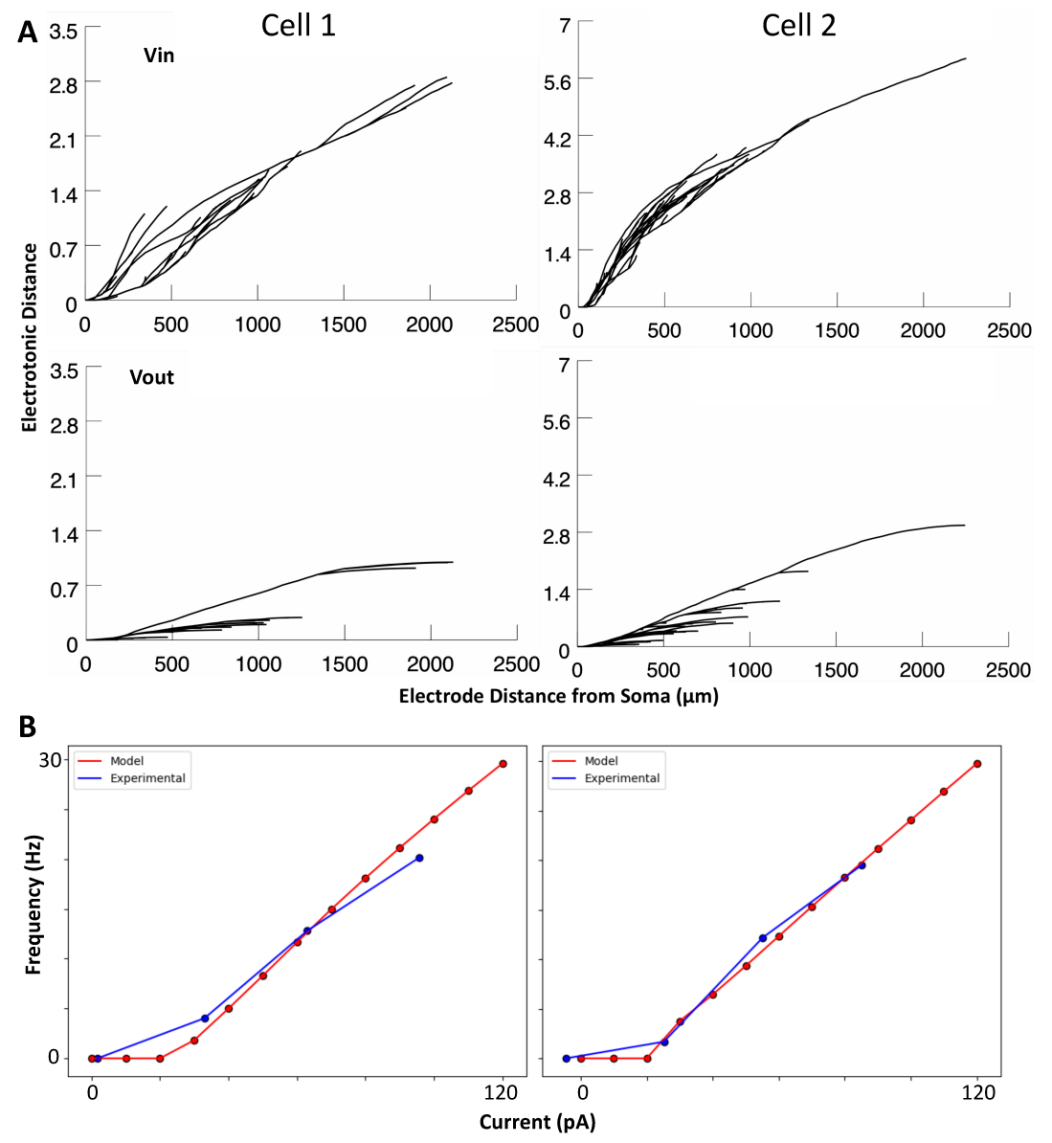
Cell 2 is more compartmentalized than cell 1, and F-I relationship is linear past rheobase. **A**: Electrotonic distance [i.e. decay of a 1 mV signal; log(voltage upstream/voltage downstream)] for voltage flowing into the soma (Vin; top) and voltage flowing out of the soma (Vout; bottom). **B**: Relationships between holding current and resulting spike rates in cell 1 and 2 models *in vitro* (red), as compared to the experimental data upon which they were optimized (blue).

We use two previously developed multi-compartment models of OLM cells (i.e. cell 1 and cell 2) ^47^. Each model was developed using a morphological reconstruction and electrophysiological dataset obtained from the same cell, including *I _H_* current recordings, which predicted that models with somatodendritic *I _H_*, rather than just somatic *I _H_*, best matched the data. Thus, the passive and active properties of each of the two models are specific to cell 1 or cell 2 and are not identical. For cell 1 and cell 2, the surface areas are 29,378.1 μm ^2^ & 35,158.5 μm ^2^, the input resistances are 360.1 MΩ & 490.2 MΩ, and the membrane time constants are 22.6 ms & 32.0 ms, respectively. Electrotonic distances are shown in Figure 1A, and for example, we note that cell 2 exhibits electrotonic distances that are up to twice as large as those seen in cell 1 ( Figure 1A) indicating more signal attenuation in cell 2. These models were developed in NEURON ^53^ and codes for the models are available on https://github.com/FKSkinnerLab/OLMng. Simulations performed for the present paper are run using NEURON 7.5 as a module in Python 2.7.

Ion channel types in our OLM cell models include: hyperpolarization-activated cyclic nucleotide-gated ( *H*), transient sodium ( *NaT*), fast and slow delayed rectifier potassium ( *Kdrf*, *Kdrs*), A-type potassium ( *KA*), M-type potassium ( *M*), T- and L-type calcium ( *CaT*, *CaL*), and calcium-dependent potassium ( *KCa*) channels. Equations describing them are given in the Appendix of 43, but specifically for *H* and *NaT* channel mechanisms, they are given in 47. Maximal conductances in soma ( *s*), axon ( *a*) or dendrites ( *d*) are represented respectively as *G _H_*, *G _NaT_*, *G _Kdrf_*, *G _Kdrs_*, *G _KA_*, *G _M_*, *G _CaT_*, *G _CaL_*, *G _KCa_*, as given in Table 1. Note that *I*_{ *channel*}_ is used to refer to the corresponding ion channel currents of the various ion channel types. Other parameters for activation, inactivation and time constants are given in 43 or 47 as specified above.

**Table 1.** Location and conductance values for ion channel types.

*Conductance* *type*	*Distribution* *location*	*Cell 1* *G (pS/µm* ^2^ *)*	*Cell 2* *G (pS/µm* ^2^ *)*
*G*_*NaT*, *s*_	soma	70.99	75.09
*G*_*NaT*, *d*_	dendrites	99.48	64.68
*G*_*NaT*, *a*_	axon	66.42	140.89
*G*_*Kdr f*, *s*_	soma	115.47	91.15
*G*_*Kdr f*, *d*_	dendrites	50.49	52.52
*G*_*Kdr f*, *a*_	axon	155.97	144.03
*G*_*Kdrs*, *s*_	soma	0.0054	0.0070
*G*_*Kdrs*, *d*_	dendrites	0.0038	0.0062
*G*_*Kdrs*, *a*_	axon	0.0082	0.0024
*G*_*KA*_	soma, dendrites	76.08	110.18
*G*_*Ca L*_	dendrites	47.19	22.01
*G*_*CaT*_	dendrites	1.01	3.74
*G*_*KCa*_	dendrites	0.14	7.08
*G*_*M*_	soma, dendrites	0.14	0.18
*G*_*H*_	soma, dendrites	0.1063	0.0608

### Synapse model

For the synapse model, we use NEURON’s built-in Exp2Syn function, which models synaptic current as a two-state kinetic scheme.

$$\begin{array}{c} i=G(V-E)\;\\ G=weight\times factor\times \left(\exp(-\frac{t}{\tau_{d}})-\exp(-\frac{t}{\tau_{r}})\right)\;(1) \end{array}$$

Where *i* is the synaptic current, *G* is the maximal synaptic conductance, *E* is the reversal potential, *V* is the membrane potential, *weight* is the synaptic weight, *factor* is a NEURON process that is used to normalize the peak synaptic conductance to the value of weight, *t* is time, *τ _r_* is the rise time, and *τ _d_* is the decay time.

### Target input populations to OLM cell models

The input populations to OLM cells that we model include interneuron specific 3 (IS3) cell inputs, GABAergic long-range projecting inputs from medial septum (MS), bistratified (BIS) cell inputs, and local pyramidal (PYR) cell inputs, and, in the absence of specific constraints, distribute them randomly across all dendritic compartments. We focus on these input populations because enough details of EPSCs and IPSCs onto OLM cells have been previously reported in the literature (IS3 cells & MS: see 33; BIS & PYR cells: see 32).

Focusing first on the IS3 cell and MS inputs ^33^, we note that these were recorded under minimal stimulation using voltage-clamp of OLM cells at +10 mV. Note that there is a junction potential correction of +15.4 mV (personal communication from L. Topolnik, Laval University, QC) such that the holding current is actually nearer to -5.4 mV. This is near the reversal potential of excitatory synapses (0 mV) and so would silence current generated from excitatory synapses, leaving only minimally evoked IPSCs from IS3 cell or MS inputs. For BIS and PYR cell inputs to OLM cells ^32^, these were not recorded under minimal stimulation (and so these currents could be generated from multiple synaptic inputs) and only the holding potential for recording excitatory inputs is reported (-60 mV; i.e. holding potential while recording BIS cell inputs is not reported). The target features of the EPSCs and IPSCs reported in the literature are summarized in Table 2.

**Table 2.** Target EPSC and IPSC features for inputs to OLM cells.

Input Type	Amplitude	Rise Time	Decay Time	Reference
IS3	13.9 *±* 2.0 pA	*1.6 *±* 0.2 ms	12.0 *±* 0.9 ms	33
MS	23.0 *±* 2.3 pA	*1.1 *±* 0.2 ms	12.1 *±* 1.1 ms	33
BIS	**16.9 pA	***1.35 ms	***12.05	32
PYR	****-12.14 pA	*****2.4 ms	*****12.7 ms	32

*20–80% rise time of IPSC **Peak IPSC amplitude is 67.6 ± 7.8 pA, but this is not with minimal stimulation. If assuming 4 synapses per connection (as per in 51), this means 16.9 pA per synapse. ***Not reported, so the values chosen are midway between the values for IS3 and MS inputs since the BIS amplitude falls about midway between the two. ****Peak EPSC amplitude is -109.3 ± 8.7 pA, but this is not with minimal stimulation. If assuming 9 synapses per connection (as per in 51), this means -12.4 pA per synapse. *****Not reported, so values used are those that were reported for excitatory inputs from Fimbria Fornix.

Also note that *E _rev_*_, *exc*_ is assumed to be equal to 0 mV and that *E _rev_*_, *inh*_ should be -87.1 mV, if taking directly from what is reported experimentally with liquid junction potential correction ^33^. For *E _rev_*_, *inh*_, if assuming that there is voltage decay from the soma to the synapse when measuring reversal potential ^29^, one needs to take a more depolarized value, so we use *-80 mV* as the inhibitory reversal potential instead. We note that there are other types of inputs to OLM cells that have been reported in the literature (e.g., serotonergic receptors ^54^, metabotropic glutamate receptors ^55, 56^, cholinergic receptors ^57– 59^, additional complexities in NMDA/AMPA/Kainate receptors ^60, 61^, and TRPV1 receptors ^62^), but in the absence of particular constraints, we opted to not include them at this time.

### Synaptic optimizations

We set the rise and decay time constants to those reported in the literature ( Table 2) and we optimized the synaptic weight parameters. Here, we describe the program that was written to perform this task. Incrementally, for each compartment, we increase the weight until the amplitude of the synaptic current that is generated is approximately equivalent to the target value obtained from the literature. Since past a certain distance from the soma, the electrotonic distance can create an exponential increase in the synaptic weight needed to generate large enough current amplitudes ^29^, we simply stop the optimizations after 100 iterations of increasing the weight. From our previous experience, letting the algorithm optimize to larger weights than this simply generates synaptic conductance predictions outside of the realm of reality when considering single-receptor conductance values and the maximum numbers of receptors per synapse seen experimentally ^29^. During these optimizations we assume that all voltage-gated channels are blocked and we set the leak reversal potential to the voltage-clamped holding potential of the model (0 mV when fitting IPSCs, and -60 mV when fitting to EPSCs). Following the optimizations, the synaptic conductances for each input type have increasing values with distance from soma ( *G _PYR_* = 0.00020 to 0.00082 *µ*S; *G _MS_* = 0.00024 to 0.00132 *µ*S; *G _IS_*_3_ = 0.00018 to 0.00068 *µ*S; *G _BIS_* = 0.00021 to 0.00100 *µ*S), most likely due to the effects of electrotonic decay.

### Generating *in vivo*-like states

In previous work using IS3 cell multi-compartment models ^51^, we performed high-resolution parameter searches in parallel on the Neuroscience Gateway (NSG) for high-performance computing ^63^ to find input parameter combinations (i.e. numbers of excitatory and inhibitory synapses and incoming spike rates) that could generate *in vivo*-like (IVL) states. We applied a similar methodology here for creating IVL states for the OLM cell models.

As done previously ^51^, we range excitatory spike rates from 0 to 30 Hz (resolution: 5 Hz), and inhibitory spike rates from 0 to 100 Hz (resolution: 10 Hz). We estimate maximal ranges for excitatory and inhibitory synapses based on findings from 64, scaled by the lengths and numbers of compartments in the OLM cell models (cell 1: 0 to 4641 excitatory synapses and we use a resolution of 35 synapses, 0 to 1989 inhibitory synapses with a 24-synapse resolution; cell 2: 0 to 6012 excitatory synapses with a 45-synapse resolution, 0 to 3006 inhibitory synapses with a 36-synapse resolution). Also, for every addition of inhibitory synapses, one third are assigned as IS3 cell inputs, another third are assigned as MS inputs, and a final third are assigned as BIS cell inputs (i.e. 8 IS3, 8 MS and 8 BIS synapses for cell 1; 12 IS3, 12 MS and 12 BIS synapses for cell 2). Note that the total numbers of synapses taken from 64 are numbers estimated for calbindin-expressing (CB+) cells. While certain subtypes of OLM cells express CB, this marker is also broadly expressed in several other cell types ^22^. As well, the CB+ morphological reconstructions shown in 64 do not appear to carry resemblances to OLM cell morphologies. Nonetheless, these estimates are still used because they are the only estimates that can reasonably be linked to synaptic densities in OLM cells. We note that when synapses are added we assume common inputs (i.e. each presynaptic spike train is assigned to multiple synapses, that is, akin to cells forming multiple synaptic connections between each other - we use 7 excitatory synapses per connection and 8 inhibitory synapses per connection for cell 1, and 9 excitatory synapses per connection and 12 inhibitory synapses per connection for cell 2).

To identify IVL states using simulated voltage recordings, we use a previously designed IVL metric ^51^ - see Equation 2. This metric uses threshold values based on experimental values found in the literature to establish whether the average subthreshold membrane potential ( ${\bar{V}}_{m}$), the standard deviation of the subthreshold membrane potential ( *σ _V _m__*), and the interspike interval coefficient of variation ( *ISICV*), are large enough for a given state to be considered IVL ^12, 14, 15, 48, 65, 66^. It also uses the average spike amplitude ( ${\bar{S}}_{A}$), to establish whether the model is entering an overly-excited state of depolarization block. Here we use a depolarization threshold value ( ${\bar{V}}_{m}$; -70.588 mV) tailored to the resting potential of OLM cells and assuming an approximate 3 mV shift *in vivo*. We used this assumption in previous work ^51^ and it is based on the shift in baseline voltage seen in CA1 place cells *in vivo* during place field traversals ^14^. We also add a spike rate criterion to ensure that the spike rate is between 3 and 25 Hz since this is known for OLM cells *in vivo* ^17, 18^. The threshold values for membrane potential standard deviation ( *σ _V _m__*), interspike interval coefficient of variation ( *ISICV*), and spike amplitude ( *S _amp_*) are the same as the values used previously for IS3 cell models ^51^.

$$\begin{array}{l} IVL\;Metric=({\bar{V}}_{m}>-70.588\;mV)+(\sigma_{V_{m}}>2.2\;mV)\\ \;\;\;\;\;\;\;\;+(ISICV>0.8)+(3\;Hz<f<25\;Hz)\\ \;\;\;\;\;\;\;\;\;\;\;\;\;\;\;\;\;\;\;\;\;\;\;\;\;\;\;\;\;-5\times (S_{amp}<40\;mV)\;(2) \end{array}$$

Given this, an IVL metric score of 4 would indicate that the input parameter combination produces an IVL state. Our parameter search yielded a variety of different excitatory/inhibitory input solution sets that could generate IVL states. As well, we observed an input parameter distribution towards low values across all input parameters, and more IVL scenarios were generated whilst in inhibitory-dominant regimes (not shown).

***Choosing in vivo-like (IVL) states.*** We chose representative IVL states from those generated by running ten randomized (i.e., of synaptic locations and presynaptic spike times as done previously ^51^) simulations for each set of input parameters that generated an IVL state, until one was found to be consistently IVL. A state is considered to be consistently IVL if the IVL metric is 4 in at least five out the ten simulations, and the other five simulations have IVL metrics of at least 3. We focused on sampling from a subset of the parameter space with low inputs (i.e., sparse enough) so as to allow cells to have larger input resistances and be more sensitive to additional rhythmically-timed inputs as previously found to be required ^51^.

In running the full parameter searches and then choosing representative IVL states from the low input subset of the parameter space we found that the amount of input was still far larger than numbers of inputs seen previously in our other models ^51^, corresponding to a more reduced input resistance and therefore a reduced sensitivity to additional inputs. To find even sparser input parameter sets that produce IVL states we sampled at a finer resolution (i.e. at a resolution of 1 synapse for numbers of synapses and 1 additional spike for spike rates) from low input parameter value sets that generate similar excitatory/inhibitory balances to the IVL states found in the full parameter search. This balance is as given by our EI Metric calculation of Equation 3 (i.e. cell 1: -42685 to -8785 *synapses × Hz*; cell 2: -65700 to -9990 *synapses × Hz*). Once we identified parameter sets that fell within these EI metric ranges, we sampled the parameter combinations in order from the lowest total input to the highest total input (i.e. see Total input calculation in Equation 3), and simulated each of these possible combinations of input parameters until another consistently IVL state was found.

$$\begin{array}{c} EI\;Metric=N_{E}\times f_{E}-N_{I}\times f_{I}\\ \;Total\;Input=N_{E}\times f_{E}+N_{I}\times f_{I}\;(3) \end{array}$$

where *N _E_* is number of excitatory synapses, *N _I_* is number of inhibitory synapses, *f _E_* is excitatory spike frequency, and *f _I_* is inhibitory spike frequency. The rationale with this approach is that IVL states should still be generated so long as the balance between excitation and inhibition falls within the right range. Following this, we found IVL states for both models where the total input is reduced (cell 1: 12,938.6 vs. 39,250 inputs; cell 2: 14,510.5 vs 46,350 inputs), which ultimately corresponds to input resistances that would allow enough sensitivity to additional inputs, as based on our previous work ^51^. The resulting input parameter values for cell 1 are 1268 excitatory synapses firing at 1.6 Hz and 1254 inhibitory synapses firing at 8.7 Hz, and for cell 2, they are 1503 excitatory synapses firing at 1.5 Hz and 1532 inhibitory synapses firing at 8 Hz.

### Approach and data analysis

To analyze and compare how the ion channel current contributions differ under *in vitro* (‘isolated slice preparation with synapses blocked’) and *in vivo* (’behaving animal’) conditions, we use the following approach with the two OLM cell models:

1. Run the models without synapses (i.e., *in vitro* state) at two different somatic holding currents above rheobase (60 pA and 120 pA). Compute the resulting spike rate and determine the slope ( *m*) and intercept ( *b*) of the line between the two data points of spike rates. Linear extrapolation of these values from just two data points is justified since the F-I curves are fairly linear above rheobase ( Figure 1B).
2. Run the models using the input parameter set that produced the chosen IVL state with a given set of random seeds and measure the spike rate ( *f _IVL_*) of the resulting spike train. Use *f _IVL_* together with *m* and *b* calculated previously to compute the holding current ( *I _hold_*) necessary to elicit a similar spike rate in the *in vitro* state:

$$I_{hold}=(f_{IVL}-b)/m\;(4)$$

3. Choose a different set of random seeds and repeat step 2 ten times.

**Figure 2.**
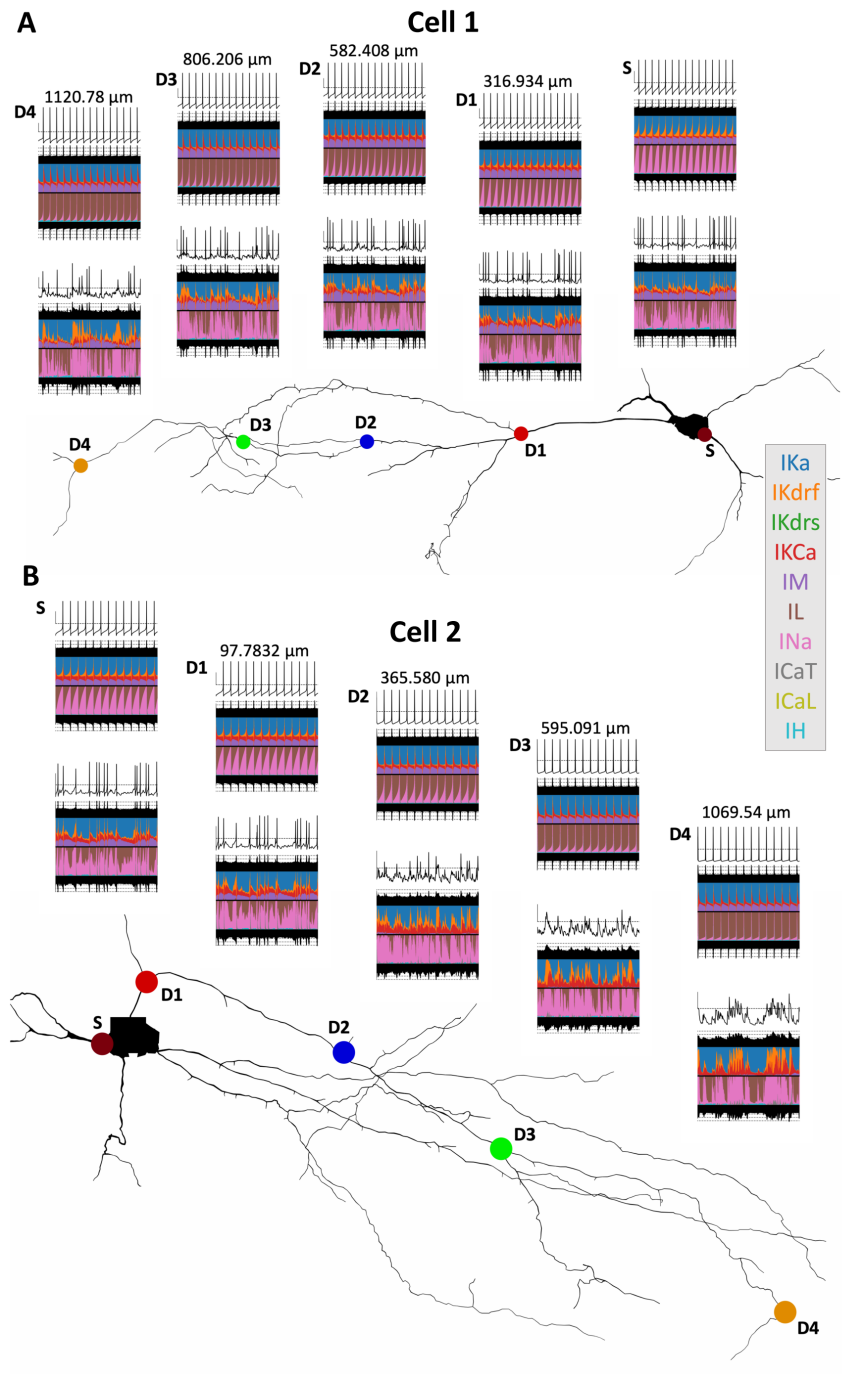
Dendritic spiking is deteriorated in the *in vivo*-like context. Currentscape plots ^67^ are shown above the shape plots in **A** (cell 1) and **B** (cell 2). The dots in the shape plots indicate the recording sites (S = Soma; D1–D4 = Dendrite 1–4). All recording sites were chosen such that they are along the same dendritic path. In each currentscape plot, the top trace is the voltage trace (y-axis scale bar = -50 mV [horizontal dashed line] to -20 mV), the filled-in black traces above and below the coloured plots are the total outward and inward currents respectively (dotted lines = ±0.5 pA, ±5 pA, ±50 pA), and the coloured plots show the percent contributions of each individual outward (top half of the plot) and inward (bottom half of the plot) current (see colour references in the legend on the right) to the total outward and inward currents. For each recording site location we show one *in vitro* currentscape plot (top) and one corresponding IVL currentscape plot (bottom), in the last second of simulation time (time axis = 9 s to 10 s). Distance values above each set of *in vitro*/IVL currentscape plots indicate the recording site distance from soma.

To consider comparisons in different locations, we choose five different recording sites in each model, which include soma and four increasingly distant dendritic compartments (i.e. soma, D1, D2, D3, and D4; Figure 2). The diameters of these locations are: 9.84 μm (soma), 1.92 μm (D1), 0.82 μm (D2), 0.94 μm (D3), and 0.75 μm (D4) for cell 1; 4.44 μm (soma), 1.26 μm (D1), 1.01 μm (D2), 0.74 μm (D3), and 0.60 μm (D4) for cell 2.

To analyze the total area under the current traces we use the numpy.trapz(current_trace,time_vector) function in Python. For outward currents, more positive values indicate larger currents, and for inward currents, more negative values indicate larger currents.

To compute cross-correlations between two time series (i.e. currents and/or voltage traces; a1 and a2), we first normalize the signals in order to generate cross-correlation magnitudes between -1 and 1, as follows:


a1 = (a1 - numpy . mean (a1)) / ( numpy .std (a1)
     * len(a1))



a2 = (a2 - numpy . mean (a2)) / numpy . std (a2)
xcorr = numpy . correlate (a1 ,a2 , mode =’full ’)


Note that all inward currents are reversed in polarity for these cross-correlations (i.e. from negative to positive), since their “activation” is reversed with respect to the polarity of voltage and outward current activation. This step allows us to better interpret the cross-correlation plots. Note that *I _L_*, though mostly an inward current at baseline, is not reversed in polarity since it becomes an outward current during spikes. Analyses of total area under the current traces and cross-correlations across current and/or voltage traces are done using the last 9 seconds of 10 second-long simulations.

To visualize the contribution of the different ion channel mechanisms to the voltage dynamics, we take advantage of currentscape plots (e.g. Figure 2), a recent visualization technique that plots the percent current contributions to the total inward or outward currents ^67^. Additional relevant code for running simulations and plotting the results shown in this paper is available online at https://github.com/FKSkinnerLab/OLM_IVLCurrents.

## Results

While it is clear that the intense synaptic bombardment present *in vivo* relative to quiescent *in vitro* states changes a cell’s response, conferring advantageous computational properties ^48^, how the different ion channel types present in different locations contribute has not been explored. In creating *in vivo*-like (IVL) states for our computational OLM cell models, as described in the Methods, we are now in a position to compare differences between *in vitro* and IVL states from the perspective of ion channel currents in somata and dendrites. As well, we can explore how the different ion channel mechanisms might contribute to cell excitability *in vivo*.

### Dendritic ion channel current contribution profiles change substantially during IVL states relative to *in vitro* states

**Figure 3.**
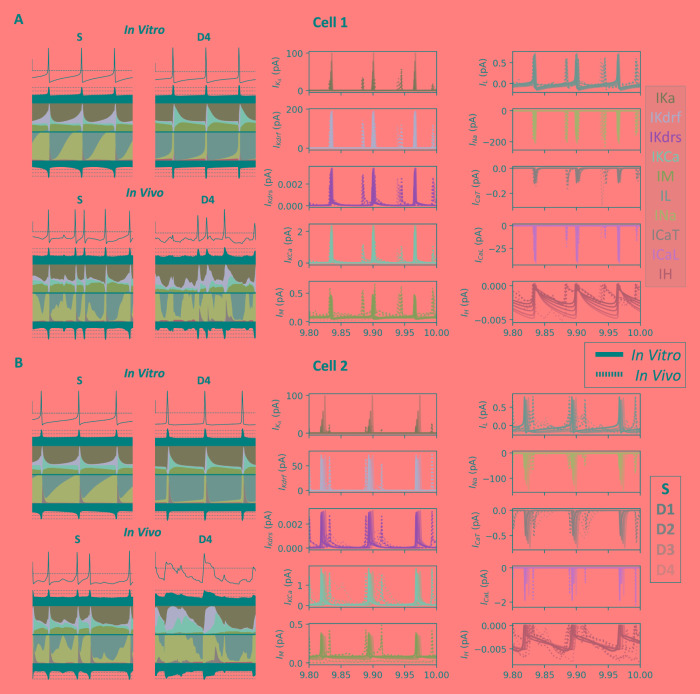
Example current recordings. Recordings show the last 200 milliseconds of a ten second simulation from an example pair of IVL and *in vitro* simulations for cell 1 ( **A**) and cell 2 ( **B**) with currentscape plots on the left (S and D4 only) and current traces on the right (IVL: dotted lines; *in vitro*: solid lines). The colour code for the currents is the same as the legend in Figure 2 but fainter coloured lines show current traces from further dendritic compartments from the soma (S to D4).

To ensure that comparable firing rates exist in the *in vitro* and IVL states of the models, we inject an appropriate amount of current into the soma of the *in vitro* models (i.e., OLM cell models without any synaptic inputs), as described in the Methods. The ion channel current contribution changes between *in vitro* and IVL states for cell 1 and cell 2 are shown in Figure 2 A and B respectively. These changes are shown in somatic compartments as well as across dendritic compartments at locations specified above the plots and as indicated in the reconstructed cell schematics. This is shown through the use of currentscape plots ^67^ where each color represents the percent of the total inward or outward current that each channel contributes across time. In the *in vitro* cases, across both models there is a shift in the *I _Na_*/ *I _L_* balance (pink/brown in Figure 2) with distance from soma, where in more distal dendritic compartments *I _Na_* contributions become narrower. However, once moved to the IVL scenario, this shift in *I _Na_* contributions is no longer apparent. This is likely because somatic current injections decay with distance from soma and thus recruit *I _Na_* in distal dendrites less, whereas in the IVL state, distal dendritic compartments are bombarded directly with synaptic inputs, and thus engage dendritic *I _Na_* more directly. A similar observation can be made with the *I _Kdrf_* contributions. To see this more clearly, we plot the somatic (S) and a dendritic (D4) location on an expanded axis so that the effect of the synaptic bombardment in dendritic regions can be seen with the voltage fluctuations ( Figure 3, left). The increase in *I _Na_* (pink) and *I _Kdrf_* (orange) in dendritic locations in the IVL scenario relative to *in vitro* is very apparent, clearly due to direct synaptic inputs in dendritic regions to activate dendritic ion channels, which would not be the case *in vitro*. In particular, *I _Na_* can persist because of less inactivation, and in cell 2, more depolarized states without spiking occur.

One stark difference between cell 1 and cell 2 is that *I _M_* (purple) contributions are almost non-existent in cell 2 dendrites during IVL states. When looking at the voltage traces for those compartments where *I _M_* is not contributing, we see that spikes fail to form, with large depolarizations occurring instead. Since *I _M_* relative contributions appear largest during interspike intervals (see 43), it is not surprising that its contribution is minimized when there is no spiking and interspike intervals are not present. We further note that cell 2 has an almost two-fold larger maximal electrotonic distance than cell 1 ( Figure 1A), which helps explain why spikes propagate less in the distal dendrites of cell 2 relative to cell 1.

In general, it is clear from looking at the voltages and current contributions that dendritic compartments are more de-correlated from each other and the soma when the model is in an IVL state (i.e. relative to the corresponding *in vitro* state). This could partially be due to the high-conductance effects of synaptic bombardment where it suppresses input sensitivity and can drown out the magnitude of unitary inputs. As a result, these smaller amplitude signals do not propagate as far, and the different morphological compartments may appear more decorrelated from each other.

### Only *I _H_* is suppressed during spikes and consistently enhanced during *in vivo*-like (IVL) states

In looking at the ion channel current traces in a relative comparison using currentscape visualization for soma vs D4 ( Figure 3, left), and in actual values for soma and all dendritic locations ( Figure 3, right), for IVL vs *in vitro* both cell 1 and cell 2, it is clear that all ion channel types become sharply activated during spikes, except for *I _H_* (cyan) which is suppressed during spikes. *I _L_* (brown) is also different but from the perspective that it is primarily an inward current at resting potentials, but sharply transitions into an outward current, specifically during spikes. This is the case across both IVL and *in vitro* states as well as in soma and in the furthest dendritic location (D4), which makes sense given the biophysical characteristics of these channels.

**Figure 4.**
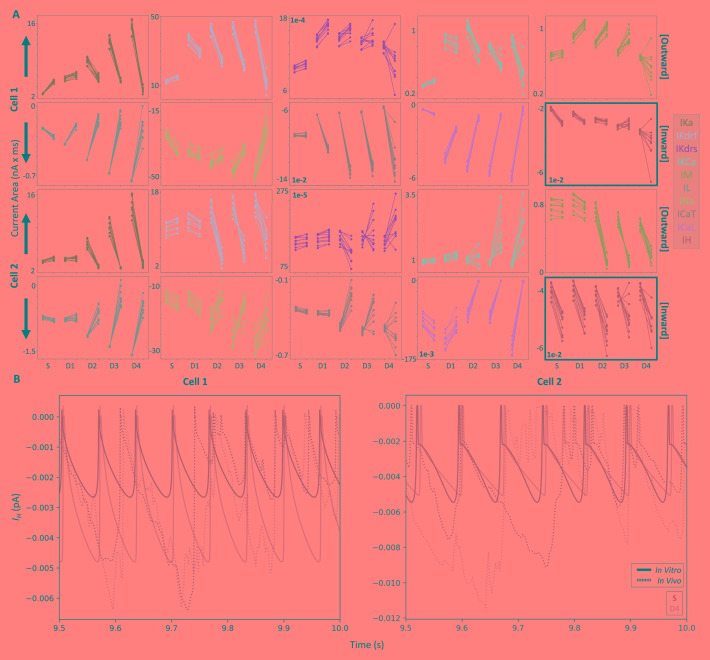
*In vitro* vs *in vivo*-like total changes in currents. **A**) Each line (10 re-randomizations = 10 lines for each recording site) connects *in vitro* results (left dots) with their corresponding IVL results (right dots). In each plot, from left to right along the x-axis, we plot soma, D1, D2, D3, and D4 (same recording sites shown in Figure 2). Note that outward currents are shown in the top rows (larger contributions = more positive values) and inward currents are shown in the bottom rows (larger contributions = more negative values). As such, the arrows indicate the direction of larger current magnitudes for each row of currents. Also note that the *I _H_* plot is highlighted with thicker borders. **B**) Example of *I _H_* traces from the S (darker cyan) and D4 (lighter cyan) compartments during the last 500 ms of IVL (dotted lines) and *in vitro* (solid lines) simulations.

In looking at changes in the total current (i.e. area under the ion channel current traces; Figure 4A) across the two models, four currents changed differently in cell 1 and cell 2 going from the *in vitro* to the IVL state. These include *I _CaT_* (increases dendritically in cell 1, but decreases dendritically in cell 2), *I _M_* (increases in cell 1, but decreases dendritically in cell 2), as well as *I _KCa_* & *I _Kdrs_* (decrease dendritically in cell 1, but increase in cell 2). We already observed and discussed this difference in *I _M_* contributions changes in the previous section (i.e. the currentscape plots in Figure 2), and it is possible that the other three ion channel currents show this difference for similar reasons (i.e. a larger suppression of dendritic spike propagation in cell 2 during IVL states), since these differences are not readily observable in the somatic compartments Figure 4A).

Across both models, many ion channel currents during IVL states relative to *in vitro* states also showed increased contributions near the soma but decreased contributions in the dendritic compartments, including *I _KA_*, *I _L_*, *I _Kdrf_*, *I _Na_*, *I _CaL_*. The only ion channel current that consistently showed increased contributions during IVL states across both models and all compartments was *I _H_*. This finding is not altogether surprising because *I _H_* is more active in subthreshold voltage ranges and further activated by inhibitory currents. To show *in vitro* and IVL state differences of *I _H_* more directly, we plot. *I _H_* from somatic and D4 compartments in Figure 4B, and the increase in total current becomes clear. That is, because the dendritic membrane potential is both bombarded by synaptic inputs and spends more time in the subthreshold voltage range relative to *in vitro* (such voltage differences can be seen in Figure 3, left), *I _H_* is more activated in dendrites during IVL states.

### Timing of ion channel current activation relative to voltage

**Figure 5.**
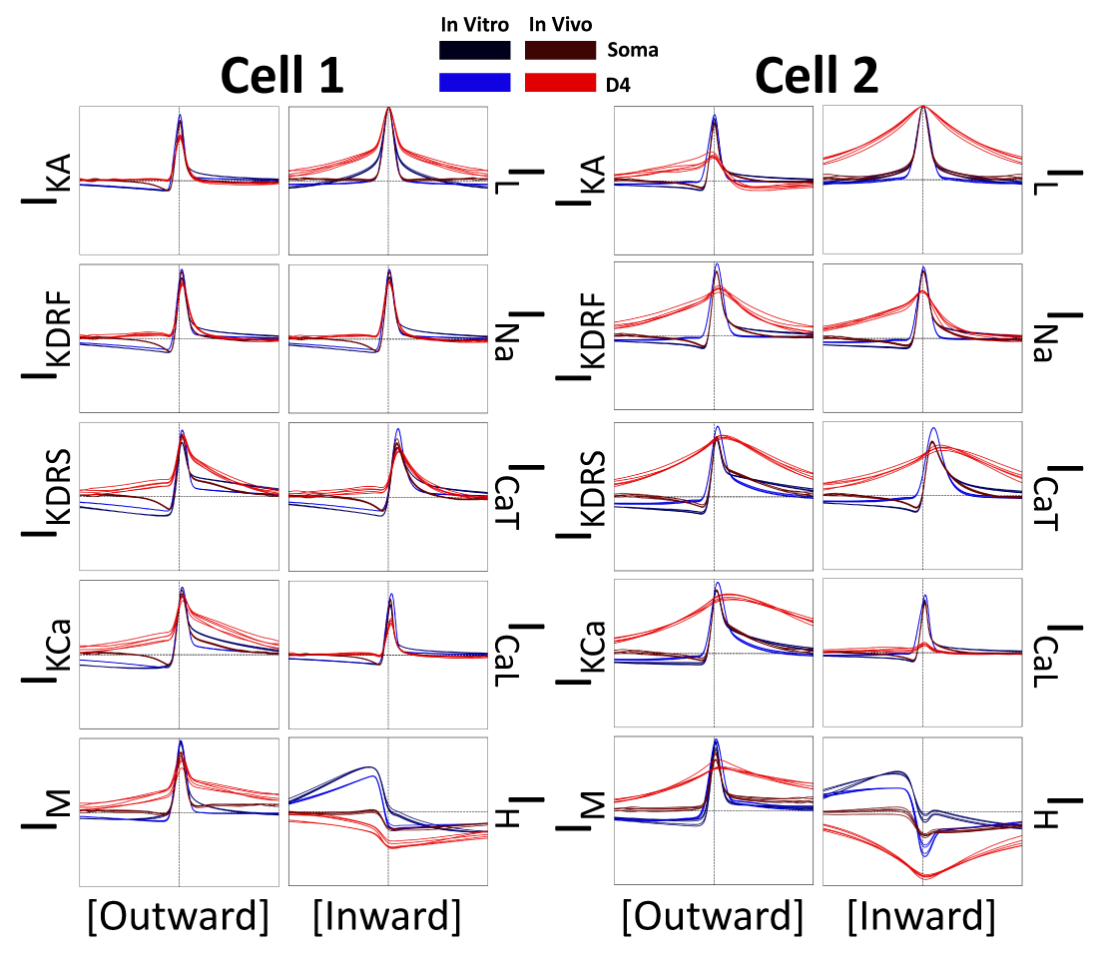
Timing of currents relative to voltage. Each plot shows the cross-correlations between the current traces and voltage traces using the last 9 seconds of each 10s trace. Note that all inward currents (right columns) are reversed in polarity for these cross-correlations (i.e. from negative to positive) in order to better interpret their activation periods with respect to voltage activation periods. Also note that we did not reverse the polarity for *I _L_* since it becomes an outward current during spikes ( Figure 3). For both cell 1 and cell 2 we run this analysis across 5 of the 10 re-randomizations of the IVL (red) and *in vitro* (blue) simulations, as well as across the somatic (darker tone) and D4 (lighter tone) compartments (see legend). The vertical and horizontal dashed lines in each plot are the zeroth axes (x-axis = -20 ms to 20 ms; y-axis = -1 to 1).

Below, we show results from analyzing the timing of ion channel currents using cross-correlations between each current trace and the corresponding location-specific voltage trace (i.e. soma or D4; Figure 5). Across all channel types, with the exception of *I _H_*, there were large narrow peaks with timescales in line with the duration of action potentials. Moreover, the lag time of the peaks are consistent across IVL vs *in vitro* states, as well as in soma vs dendrites, indicating that the relative timing of different ion channel currents is unchanged whilst under synaptic bombardment. Also across all dendritic ion channel currents in the IVL state, the cross-correlation timescale becomes broader, likely due to the general loss of spikes in more distal dendrites and longer timescale plateau-like potentials (i.e. as in the voltage trace plots in Figure 2). Some of the ion channel currents that were tightly linked with spiking had slightly non-zero peak lag times, indicating delayed activation (e.g. *I _Kdrf_* / *I _Kdrs_* / *I _KCa_* showing delayed rectification properties and *I _CaT_* / *I _CaL_* becoming more active in the after-spike hyperpolarization period). Others had peaks that were centered almost exactly at zero ( *I _K _A__*, *I _Na_*, *I _M_*, and *I _L_*, which could all contribute to balancing action potential amplitude). In particular, *I _L_* and voltage cross-correlations were almost entirely symmetrical to the point where they appeared to be auto-correlations, indicating that *I _L_* is a good proxy for gauging changes in voltage. Finally, *I _H_* was unique in that it is the only ion channel current type that exhibited a negative cross correlation with spiking, which is in line with h-channels being activated during hyperpolarization and not spiking. Moreover, the cross-correlation between *I _H_* and voltage is even more negative in dendrites relative to soma, and during IVL states relative to *in vitro* states. This suggests that *I _H_*, which overall is enhanced during IVL states ( Figure 4), is comparatively more suppressed during spikes because the relative change in *I _H_* during a spike is larger in the IVL state.

### Timing of ion channel current activations relative to each other

**Figure 6.**
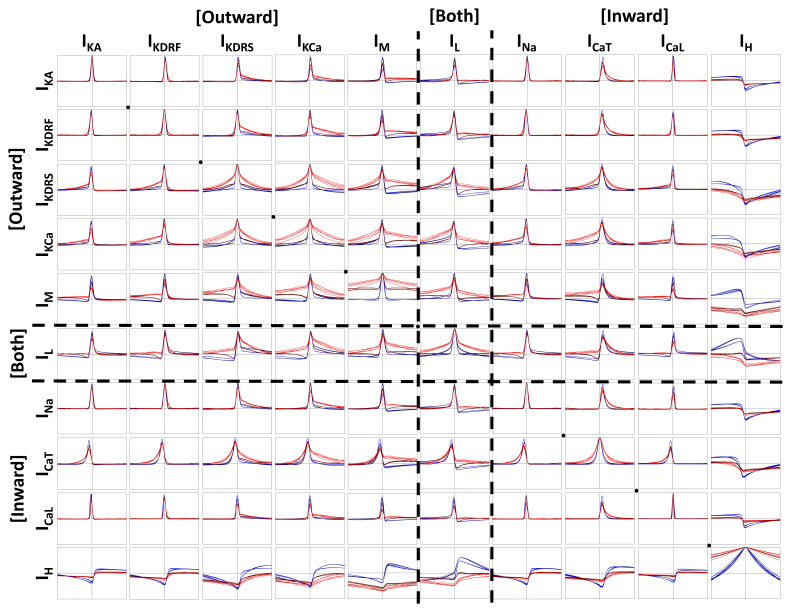
Timing of currents relative to each other (cell 1). Cross-correlations are plotted in the same way as in Figure 5 (i.e. same legend but different cross-correlation pairs). The diagonal black dots, highlight channel auto-correlations (e.g. *I _K _A__* * *I _K _A__*). Everything above the diagonal is cross-correlated one way and everything below the diagonal is cross-correlated the opposite way (i.e. like a mirror; e.g. I _K _A__ * I _Kdrf_ vs. *I _Kdrf_* * *I _K _A__*). The vertical and horizontal dashed lines in each plot are the zeroth axes (x-axis = -20 ms to 20 ms; y-axis = -1 to 1).

**Figure 7.**
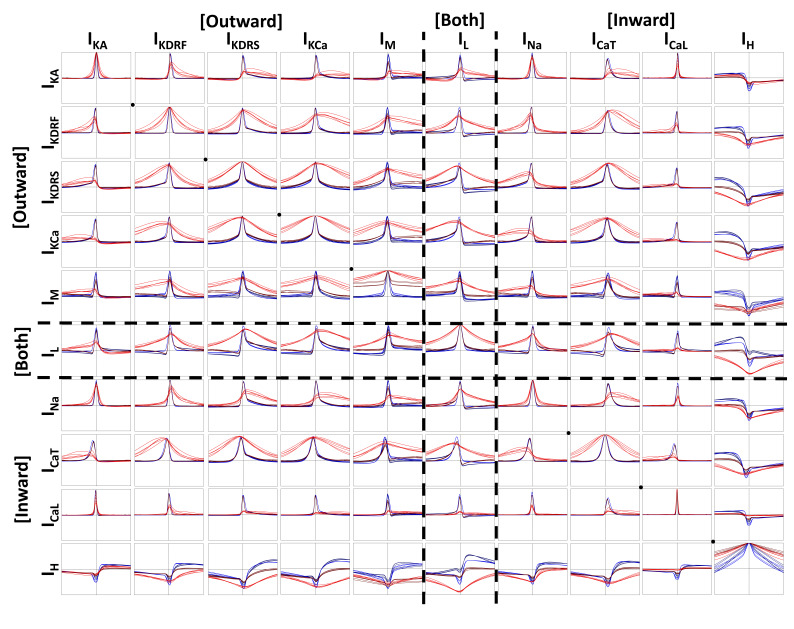
Timing of currents relative to each other (cell 2). Cross-correlations are plotted in the same way as in Figure 5 (i.e. same legend but different cross-correlation pairs). The diagonal black dots, highlight channel auto-correlations (e.g. *I _K _A__* * *I _K _A__*). Everything above the diagonal is cross-correlated one way and everything below the diagonal is cross-correlated the opposite way (i.e. like a mirror; e.g. I _K _A__ * I _Kdrf_ vs. *I _Kdrf_* * *I _K _A__*). The vertical and horizontal dashed lines in each plot are the zeroth axes (x-axis = -20 ms to 20 ms; y-axis = -1 to 1).

Below, we show cross-correlations between every possible ion channel current combination in order to examine co-activation relationships ( Figure 6 and Figure 7). Across all cross-correlations, regardless of state, cell, or recording site, the peak lag time is preserved. While almost all cross-correlations exhibited peaks aligned very near zero, only *I _CaT_* consistently exhibited non-zero lag time peaks, indicating a delay in *I _CaT_* activation relative to other ion channel current activations. More specifically, most currents activate very close to when spikes are occurring ( Figure 5), but *I _CaT_* appears to exhibit a considerable delay relative to the timing of the other currents, and is more likely aligned to when the cell is undergoing after-spike hyperpolarization.

Also, of note, is the particular asymmetrical shape of any of the cross-correlations with *I _H_*. *I _H_* exhibited negative cross-correlation peaks with all of the other ion channel currents (i.e when the other currents increase in magnitude, *I _H_* decreases in magnitude). Specifically, this translates to *I _H_* showing decreased contributions during spikes, while all of the other ion channel currents show enhanced contributions during spikes ( Figure 5). The shape of *I _H_* cross-correlations are likely a result of the fact that *I _H_* possesses a longer and generally asymmetrical time course relative to the time course of currents that activate most strongly during spikes ( Figure 3).

When looking at differences between IVL (red) and *in vitro* (blue) states and across cellular compartments, we see that the amplitude of the peak cross-correlation is dependent on the cell ( Figure 6 and Figure 7). For example, with cell 1 ( Figure 6), where spiking is more easily propagated across dendritic compartments ( Figure 2A), we see that the ion channel currents that are activated during spiking do not show much change in the amplitude of the peak cross-correlations. However, for cell 2 ( Figure 7), where spiking is less easily propagated across dendritic compartments ( Figure 2B), the peak cross-correlations are decreased and broader in the IVL states. This was particularly the case for dendritic compartments, which exhibit broader cross-correlation peaks. Moreover, the somatic IVL cross-correlations are often closer in shape to the *in vitro* cross-correlations than they are to the dendritic IVL cross-correlations. One notable exception to this are the cross-correlations of either *I _M_* or *I _L_* with *I _H_*. In all cases, these cross-correlations with *I _H_* have more negative peak magnitudes whilst in IVL states (across both soma and dendrites), which parallels the cross-correlations between *I _H_* and voltage ( Figure 5). This may be related to the *I _H_* traces consistently being enhanced across compartments during IVL states ( Figure 4). As well, across many cross-correlations with *I _H_* (i.e. *I _Kdrf_*, *I _Kdrs_*, *I _KCa_*, *I _M_*, *I _L_*, *I _Na_*, and *I _CaT_*), IVL peak cross-correlations appear to be more negative in dendritic compartments than somatic compartments. This is in sharp contrast with other IVL state cross-correlations, which generally all exhibit larger cross-correlation peaks in somatic locations. This observation is seen more clearly with the cell 2 plots ( Figure 7). In summary this work predicts that, *in vivo*, dendritic *I _H_* is enhanced and as such, can be suppressed by a larger degree whenever other channels are more active. At the same time, this suggests that other dendritic ion channel currents show weaker co-activation between each other *in vivo*.

Altogether this suggests that *I _H_* activation is more prominent in dendritic compartments than in somatic compartments, as well as during IVL states where dendritic *I _H_* is more directly activated through synaptic bombardment. Comparatively, cross-correlations between other dendritic ion channel currents that are more active during spikes will have decreased co-activation during IVL states, potentially because of increased compartmentalization and a resultant decrease in the propagation of spikes across compartments.

### Morphological compartments are more decorrelated with each other during *in vivo*-like states

**Figure 8.**
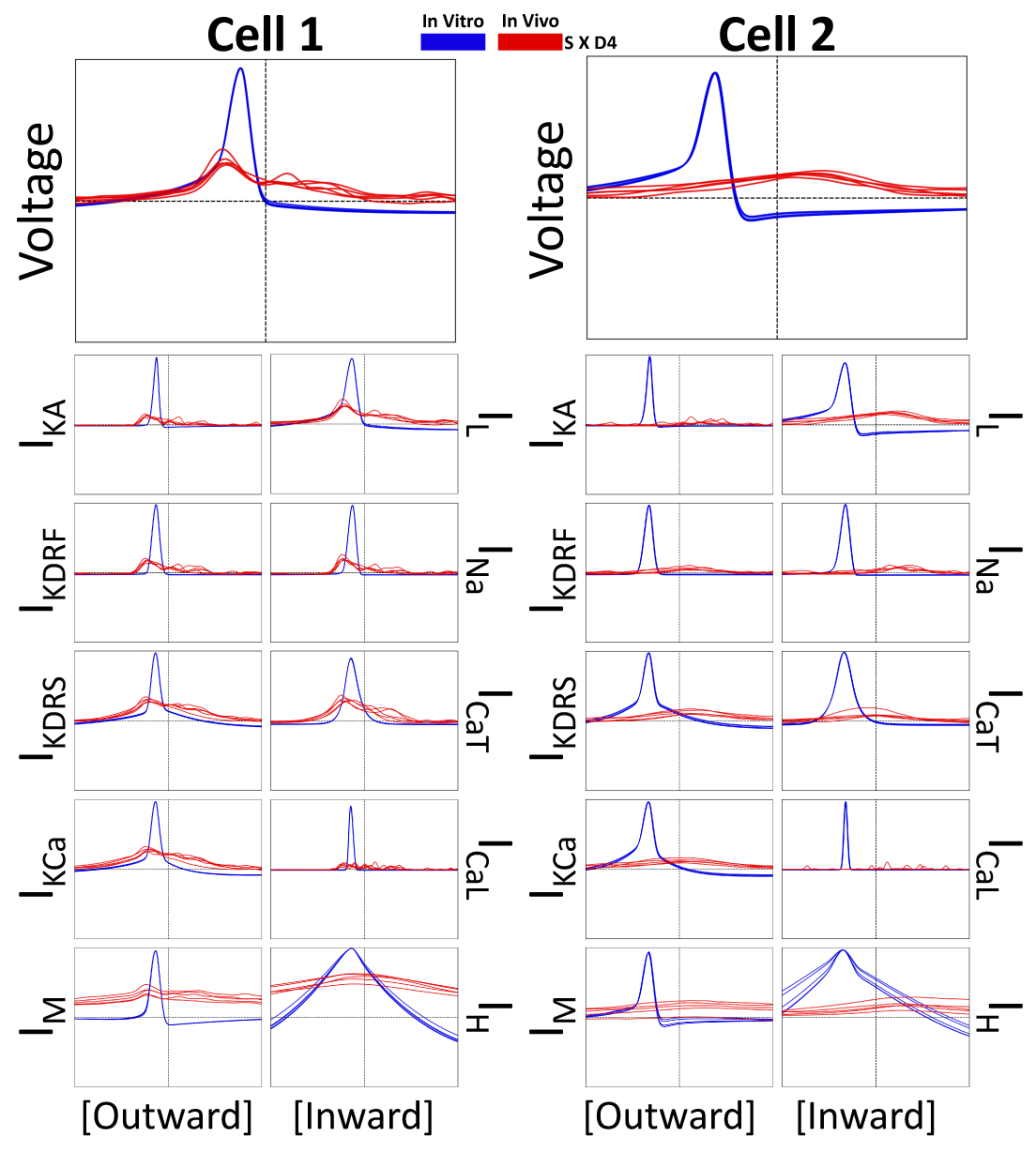
Relative timing of voltage and currents across soma and D4. Each plot shows cross-correlations ( *in vitro* = blue; IVL = red) of a somatic current type or voltage cross-correlated against the same current type or voltage recorded in the D4 compartment. Cross-correlations are plotted in the same way as in Figure 5.

Having observed a decrease in the dendritic propagation of spikes in the IVL context ( Figure 2), we can hypothesize that morphological compartments will become more de-correlated with each other during these states. As such, we further analyze channel current cross-correlations between the voltage and current traces in the soma and the corresponding traces in the D4 compartment ( *in vitro* = blue; IVL = red; Figure 8).

It is clear from these plots that ion channel current activity is considerably de-correlated during IVL states since the *in vitro* amplitudes of the cross-correlation peaks (blue) are considerably larger than the IVL amplitudes of the cross-correlation peaks (red). In the previous currentscape plots ( Figure 2) showing the *in vitro* cases, action potentials recorded in D1-D4 appeared to be the result of back-propagating action potentials generated near the soma. Here we show that this is indeed the case since the blue maximal peaks align at negative lag times ( Figure 8).

Interestingly, there is somewhat of a difference between cell 1 and cell 2 for the IVL cases ( Figure 8). While they both exhibited very diminished peaks compared to their corresponding *in vitro* cases, the lag time of the peaks was considerably different. Cell 1 exhibited more pronounced negative lag times, suggesting that spikes were being generated near to the soma and back-propagating to the D4 distal dendrite recording site (though slightly more slowly and with a considerably diminished amplitude compared to the *in vitro* spike back-propagation). On the other hand, cell 2 exhibited positive and broader peak lag times, suggesting that dendritic postsynaptic potentials are preceding somatic spikes with minimal spike back-propagation. We already know that spikes do not back-propagate easily into cell 2’s distal dendrites due to the combination of increased electrotonic distance ( Figure 1A) and synaptic bombardment ( Figure 2B). However since there is a positive peak, albeit small, we can presume that distal synaptic input are still coherently integrated such that they can lead to spikes at the soma in cell 2 ( Figure 8).

Although broad, IVL *I _H_* cross-correlations between soma and D4 were larger than the IVL cross-correlations between morphological compartments for other ion channel currents, suggesting that *I _H_* is possibly more resistant to the de-correlating effects of synaptic bombardment, possibly due to it being more active during subthreshold periods. This is intuitive given that the dendritic compartments during IVL states are more likely to be in subthreshold regimes due to dendritic plateau/complex spiking effects brought on by synaptic bombardment ( Figure 2).

## Discussion

In this work we have computationally explored ion channel current contributions that are seen across different morphological compartments of an interneuronal cell type, the OLM cell, in *in vitro* and *in vivo*-like states. In doing so, we assessed the relative timing of ion channel current activation across pairs of ion channel types, across morphological compartments, and relative to voltage — a task that is not possible to perform experimentally *in vivo*, and that would be almost impossible to do *in vitro*. We found that the relative timing of ion channel current co-activation is preserved across states and locations. However, the magnitudes and relative contributions of the different ion channel currents are altered between states and across locations, and different morphological compartments become more de-correlated with each other during *in vivo*-like states. In particular we observe a consistent enhancement in *I _H_* across spatial compartments during *in vivo*-like states relative to *in vitro*, which could coincide with the de-correlation seen across morphological compartments.

### Insights and interpretations

In this era of big data and channelopathies, it is highly desirable to be able to know and understand how different ion channel types might contribute to normal and pathological states. For example, a link between big potassium channels and epilepsy in Angelman syndrome was recently shown ^68^. In this ’tour de force’ experimental study, genetic, organoid and behavioural platforms were used to suggest this link. Computational modeling approaches as presented here could be harnessed to make such links and hypothesize others by examining particular ion channel types in cellular and networks states that are akin to *in vitro* and *in vivo*-like/behavioural states. Interestingly, in light of our results here, recent work has shown that blockage of HCN channels in OLM cells prevented the formation of type 2 theta rhythms (that emerge during immobile, anxiety-laden behavioural states) as controlled by OLM cells in ventral hippocampus ^69^.

We had noticed an enhancement of dendritic *I _H_* during *in vivo*-like states where subthreshold depolarizations are dominant. This follows since *I _H_* is a current that is most active during subthreshold and hyperpolarization regimes where it can be further activated by inhibitory perturbations ^41, 70^. In our models, our fitting required that synaptic conductance scales up with distance from soma, which could also contribute towards further enhancing dendritic *I _H_*. This is interesting considering the differential dendritic expression of *I _H_* in different cell types. For example, in layer 5 cortical pyramidal cells ^71, 72^ and CA1 hippocampal pyramidal cells ^73^ *I _H_* scales up in the apical dendrites with distance from soma. In OLM cells, our developed models provided support for h-channels being present in dendrites, but non-uniform distributions were not specifically examined ^47^. Our previous computational studies showed that dendritic *I _H_* in OLM cells can modulate the input frequencies at which they are preferentially recruited to spike ^46^. This suggests that if differential dendritic expression of *I _H_* were present in OLM cell dendrites, it could serve as a frequency modulator. Since our study here shows that dendritic *I _H_* is enhanced during *in vivo*-like states and operates in the subthreshold regime, it is likely to play a role in subthreshold signal propagation. Previous work has mostly highlighted that *I _H_* reduces signal propagation and enhances compartmentalization of dendrites by reducing the input resistance in principal neuron dendrites across hippocampus ^74, 75^, cortex ^76^, and basolateral amygdala ^77^. Expression and function of *I _H_* is also known to be modulated by long-term synaptic potentiation (LTP) mechanisms, where LTP can upregulate HCN channels, while at the same time suppress channel function ^78^. Altogether, this suggests that enhanced dendritic *I _H_ in vivo* will contribute towards increasing dendritic compartmentalization. Since we do see a decrease in backpropagating action potentials ( Figure 2) and decreased cross-correlations between spatial compartments ( Figure 8) in the *in vivo*-like state, these may be a by-product of enhanced *I _H_*. However, it could also be due to a global effect across all of the synaptic and intrinsic currents that are enhanced in the *in vivo*-like state, which, together, all contribute towards decreasing the input resistance and suppressing the propagation of signals. We note that a more detailed sensitivity analysis may be warranted to better uncover these mechanisms, possibly using some of the visualization techniques made available through 67 to explore the full range through which currents may become rebalanced during conductance perturbations.

### Limitations

In our *in vivo*-like states we did not try to directly simulate synaptic inputs linked to any particular behavioral paradigm (e.g. rhythmic or bursting inputs during theta or sharp waves). That is, we generated *in vivo*-like states that simulate the levels of synaptic bombardment that neurons might receive *in vivo* ^12, 48^. More specifically, we highlighted changes in ion channel current contribution profiles that may exist *in vivo*, and contextualized how these changes may affect the way in which individual neurons process behaviourally-relevant information. We note that in future work it will be informative to analyse current contribution profiles during these *in vivo*-like states and in the context of sensory-evoked stimuli, which have been shown to evoke dendritic spike events in pyramidal cells ^73^ and so would be interesting to study in the context of inhibitory interneurons. 

In general, using computational simulations alone to investigate biophysical phenomena carries its own set of caveats. Though the models that we use are data driven, no model is ever truly complete ^79^, and degeneracies are to be expected ^80^. Moreover, many assumptions need to be made when constructing a morphologically-detailed multi-compartment model, such as types of ion channels to include and their distribution across the morphology of the model.

## Conclusions

In summary, this work is a computational investigation into the dendritic ion channel contributions that govern OLM cell excitability *in vivo*. We highlight that the timing of ion channel currents relative to voltage and each other are invariant across states, though many undergo changes in their current output magnitudes. In particular dendritic *I _H_* is enhanced during *in vivo*-like states, which could indicate altered signal propagation in behaving animals relative to *in vitro* recordings. Finally, we show that during *in vivo*-like states, voltage and currents across compartments become more de-correlated relative to each, with a shift in the lag time of their maximal cross-correlation peaks. This was indicative of a loss of backpropagating action potentials, which made cross-correlations between subthreshold signals and spikes more apparent. Overall, this work shows a possible way to explore and gain insight into the coordination of ion channel currents that govern neuronal spiking in the “behaving animal”.

## Data availability

### Underlying data

Code for running simulations and plotting the results: https://github.com/FKSkinnerLab/OLM_IVLCurrents

Archived code as at time of publication: http://doi.org/10.5281/zenodo.3688619 ^81^

License: GNU General Public License v3.0

Models of cell 1 and 2: https://github.com/FKSkinnerLab/OLMng

Archived models as at time of publication: http://doi.org/10.5281/zenodo.3689724 ^82^

License: GNU General Public License v3.0
