# Supplementary material for: Computationally going where experiments cannot: a dynamical assessment of dendritic ion channel currents during in vivo-like states
Source: F1000Res. 2020 Jun 11;9:180. Originally published 2020 Mar 11. [Version 2] doi: 10.12688/f1000research.22584.2 (PMC7309567; doi:10.12688/f1000research.22584.2)
Supplement: Peer review history

# Peer review: Reviewer response for version 2

**DOI:** 10.5256/f1000research.27205.r64634

**Published:** 2020-06-18

**Authors:** Fernando Fernandez

**Affiliations:** 1 Department of Biomedical Engineering, Center for Systems Neuroscience, Boston University, Boston, MA, USA

The authors have addressed my concerns.

Is the work clearly and accurately presented and does it cite the current literature?

Yes

If applicable, is the statistical analysis and its interpretation appropriate?

Yes

Are all the source data underlying the results available to ensure full reproducibility?

Yes

Is the study design appropriate and is the work technically sound?

Yes

Are the conclusions drawn adequately supported by the results?

Yes

Are sufficient details of methods and analysis provided to allow replication by others?

Yes

Reviewer Expertise:

Slice and in vivo intracellular electrophysiology, biophysical modeling of neurons.

I confirm that I have read this submission and believe that I have an appropriate level of expertise to confirm that it is of an acceptable scientific standard.

# Peer review: Reviewer response for version 1

**DOI:** 10.5256/f1000research.24930.r62251

**Published:** 2020-05-04

**Authors:** Vijayalakshmi Santhakumar, Andrew Huang

**Affiliations:** 1 Department of Molecular, Cell and Systems Neuroscience, University of California at Riverside, Riverside, CA, USA; 2 University of California at Riverside, Riverside, CA, USA

Experimental evaluation of the complex interactions between ion channels in dendritic segments is challenging and inhibitory neuron dendrites are especially difficult to assess using *in vivo *and *in vitro* patch-clamp studies. However, understanding channel interactions with synaptic inputs shape integration, AP initiation and propagation is integral to information processing in specific circuits and identifying principles that govern brain function and disease. Guet-McCreight and Skinner approach this issue by adopting detailed multi-compartmental computation models of morphologically reconstructed oriens-lacunosum-moleculare neurons (OLM cells) to examine recruitment of specific ion channels during activity. Specifically, the simulations focus on comparing channel recruitment across somatodendritic compartments under “ *in vitro*-like states” during somatic current injection in the absence of synaptic input and *“in vivo*-like state” with E-I balanced synaptic inputs. Using morphological simulations of distinct OLM cells, they highlight the specifically salient role for h-currents under *in vivo*-like conditions of heightened dendritic synaptic activity. The paper is largely descriptive, and findings are intuitive given the model design. The study provides value in demonstrating the potential application of current-scape plots and computational modeling in assessing synaptic/dendritic behavior and could be strengthened with additional consideration/discussion of the role for Ih in synaptic integration.

**Major:**

1. While it is known that several channels show gradients in somatodendritic distribution the models presented in the study assume uniform dendritic distribution of channels. Given the lack of specific data on distribution, the assumption to distribute channels uniformly is understandable. However, the discussion could speculate how differences in channel distribution may alter the overall conclusions. This is particularly relevant to h-channels which are known to have differential dendritic distributions in certain cell types.
2. The heavy inhibitory bias of *in vivo-*like inputs is consistent with experimental data. IPSCs are extremely effective in activating h-channels, as such, it is not surprising that h currents are more pronounced in the *in vivo-*like and sub-threshold conditions rather than during depolarizing current injections which would move the membrane outside the activation range for h-channels. Additionally, the dendritic synapses appear to be scaled up which could further increase h-channel activation during the IPSCs. These aspects warrant discussion.
3. Given the focus on Ih, it may make it easier for the reader if the panels representing Ih data are identified with bold borders or included in a separate figure for discussion. The activation by hyperpolarization and in the subthreshold regimen make Ih different from other channels that activate on depolarization, thus the findings are not surprising. The unique role of Ih in signal integration/ dendritic compartmentalization and how it may contribute to input resistance, the relevance of the relatively slower kinetics of Ih can be better explored.  
4. Overall, it is unclear what Figure 4 adds to the manuscript as it compares currents at very different conditions and would be heavily dependent on membrane conductance that differs greatly between these conditions.  

 

**Minor:**

1. Figure 3: the scales are very different for the *in vivo *and *in vitro *conditions even for the same parameter making it difficult to compare the differences. This is the only figure presenting absolute values and appropriate scaling would improve the ability to directly compare amplitudes.  
2. Utilization of NSG “to find input parameter combinations” is a good start but probably does not fully reflect *in vivo* activity patterns during behavior. The limitations associated with this should be discussed. Were parameters based on any specific behavior (stimulus/inputs)?
3. Approach and data analysis:
  1. apostrophe backwards in parenthesis (‘isolated…’ and ‘behaving…’)
  2. Pg. 7: “dis-tal” àdistal

Is the work clearly and accurately presented and does it cite the current literature?

Yes

If applicable, is the statistical analysis and its interpretation appropriate?

Yes

Are all the source data underlying the results available to ensure full reproducibility?

Yes

Is the study design appropriate and is the work technically sound?

Yes

Are the conclusions drawn adequately supported by the results?

Yes

Are sufficient details of methods and analysis provided to allow replication by others?

Yes

Reviewer Expertise:

Epilepsy, inhibition, brain injury, computational modeling, electrophysiology

We confirm that we have read this submission and believe that we have an appropriate level of expertise to confirm that it is of an acceptable scientific standard.

# Response

**Published:** 2020-06-06

**Authors:** Alexandre Guet-McCreight

**Affiliations:** University of Toronto, Canada

We thank the reviewers for their comments and detailed review of our manuscript. It has led to a better presentation and explanation of our work.

 We have revised our manuscript accordingly, changing and expanding two of the existing figures to exhibit results more clearly and providing more explanations. We hope that the changes made sufficiently addresses the concerns brought up by the reviewers. Please find below our specific responses (in **bold **font).

**Reviewer 2: Vijayalakshmi Santhakumar**

 Experimental evaluation of the complex interactions between ion channels in dendritic segments is challenging and inhibitory neuron dendrites are especially difficult to assess using *in vivo *and *in vitro* patch-clamp studies. However, understanding channel interactions with synaptic inputs shape integration, AP initiation and propagation is integral to information processing in specific circuits and identifying principles that govern brain function and disease. Guet-McCreight and Skinner approach this issue by adopting detailed multi-compartmental computation models of morphologically reconstructed oriens-lacunosum-moleculare neurons (OLM cells) to examine recruitment of specific ion channels during activity. Specifically, the simulations focus on comparing channel recruitment across somatodendritic compartments under “ *in vitro*-like states” during somatic current injection in the absence of synaptic input and *“in vivo*-like state” with E-I balanced synaptic inputs. Using morphological simulations of distinct OLM cells, they highlight the specifically salient role for h-currents under *in vivo*-like conditions of heightened dendritic synaptic activity. The paper is largely descriptive, and findings are intuitive given the model design. The study provides value in demonstrating the potential application of current-scape plots and computational modeling in assessing synaptic/dendritic behavior and could be strengthened with additional consideration/discussion of the role for Ih in synaptic integration.

*Major:*

 While it is known that several channels show gradients in somatodendritic distribution the models presented in the study assume uniform dendritic distribution of channels. Given the lack of specific data on distribution, the assumption to distribute channels uniformly is understandable. However, the discussion could speculate how differences in channel distribution may alter the overall conclusions. This is particularly relevant to h-channels which are known to have differential dendritic distributions in certain cell types.

**We have added a clarification to the methods that the models were developed with IH current recordings from the same cell that predicted the presence of h-channels in the dendrites, and not only in the soma.  As discussed in Sekulic et al, non-uniform distributions may be possible in OLM cells, but what that work allowed us to robustly show is that it is very likely that h-channels are present in the dendrites, and any further examinations of non-uniform distributions would require further experimental data to be able to explore.  From our various figures that show Ih along the dendritic tree and other works, we now speculate on the situation if h-channels are not uniformly distributed.  For example, different spatial Ih distributions (in combination with different spatial synaptic distributions) could contribute towards modulating the input frequency at which cells can be recruited to spike. We have also added some comparative discussion points with respect to the distribution of IH in layer 5 pyramidal cell.**

 The heavy inhibitory bias of *in vivo-*like inputs is consistent with experimental data. IPSCs are extremely effective in activating h-channels, as such, it is not surprising that h currents are more pronounced in the *in vivo-*like and sub-threshold conditions rather than during depolarizing current injections which would move the membrane outside the activation range for h-channels. Additionally, the dendritic synapses appear to be scaled up which could further increase h-channel activation during the IPSCs. These aspects warrant discussion.

**Thank you for making these comments.  We agree with these points and have now included them along with expansions to make things more clear with details and explanations, including the point that inhibitory conductances scale up with distance from soma to the discussion and added these points to the results section where increased Ih is first reported.**

 Given the focus on Ih, it may make it easier for the reader if the panels representing Ih data are identified with bold borders or included in a separate figure for discussion. The activation by hyperpolarization and in the subthreshold regimen make Ih different from other channels that activate on depolarization, thus the findings are not surprising. The unique role of Ih in signal integration/ dendritic compartmentalization and how it may contribute to input resistance, the relevance of the relatively slower kinetics of Ih can be better explored.  

**We have added thicker border lines to the IH plots in figure 4 as well as plots of IH traces in expanding Figure 4. We have also added further specific details unique to each cell model (input resistance, diameters, surface areas, etc.) so that model differences are clearer.**

 Overall, it is unclear what Figure 4 adds to the manuscript as it compares currents at very different conditions and would be heavily dependent on membrane conductance that differs greatly between these conditions.  

**We have revised Figure 3 (to show current changes along the dendritic tree and across IVL and *in vitro *contexts on the same plot).  However, as Figure 4 is showing the ‘total current’ in terms of area under the curve, we think that this is helpful in showing that Ih is the main current affected when comparing in vitro and in vivo, and to make the Ih total current more apparent, we show Ih in Figure 4B now – the increase in total current in vivo relative to in vitro can be more clearly seen, as summarized in the datapoints of Figure 4A. We agree that the results are heavily dependent on membrane conductance and this comparison is important for looking at current channel contributions and magnitudes across conditions. We are looking at the effects of in vivo-like states on ion channel current contributions and increases in membrane conductance is one of the primary differences between the in vivo-like state and the in vitro state. We note that to ensure that the comparison is reasonably ‘fair’, we used injected current values in the in vitro state to give rise to similar spike rates (at the soma) in both contexts, as described in the Methods.**

*Minor:*

 Figure 3: the scales are very different for the *in vivo *and *in vitro *conditions even for the same parameter making it difficult to compare the differences. This is the only figure presenting absolute values and appropriate scaling would improve the ability to directly compare amplitudes.  

**To facilitate comparisons, we have now moved the in vivo/in vitro and S/D1/D2/D3/D4 traces to the same plots and zoomed in to a 200 ms time range. We have also added h-current plots to figure 4 for the S/D4 compartments to facilitate comparison of these traces across conditions.**

 Utilization of NSG “to find input parameter combinations” is a good start but probably does not fully reflect *in vivo* activity patterns during behavior. The limitations associated with this should be discussed. Were parameters based on any specific behavior (stimulus/inputs)?

**We discuss this aspect in the limitations section of our discussion, and we have now extended this point. The decision for the ranges of input parameters is outlined in the section in the methods and are not based on specific sensory-evoked input paradigms.  As described in the Methods, the extensive computational exploration to determine IVL states parallels the previously used approach in Guet-McCreight & Skinner (2019) for IS3 cells.**

 Approach and data analysis:

1. apostrophe backwards in parenthesis (‘isolated…’ and ‘behaving…’)
**Fixed – thank you.**
2. Pg. 7: “dis-tal” àdistal

**Fixed – thank you.**

# Peer review: Reviewer response for version 1

**DOI:** 10.5256/f1000research.24930.r61212

**Published:** 2020-04-06

**Authors:** Fernando Fernandez

**Affiliations:** 1 Department of Biomedical Engineering, Center for Systems Neuroscience, Boston University, Boston, MA, USA

In the manuscript by Guet-McCreight and Skinner, the authors use two variants of an OLM compartmental model to probe the impact of in vivo-like synaptic activity on membrane voltage fluctuation behavior and the differential recruitment of voltage-gated conductances.

 Overall the paper provides interesting insights into how synaptic inputs differentially impact voltage-gated conductances in dendrites and the cell body. Further, the impact of in vivo-like inputs on dendritic membrane voltage dynamics is particularly interesting as it dramatically increases the timescale of fluctuations and creates “plateau-like” depolarizations. In addition, the authors show that differences within the two biologically based OLM models can lead to substantial differences in dendritic voltage behavior and that these differences are enhanced when driven by in vivo-like inputs.

**Major Issues:**

 Although many of the results noted above are interesting. It would be helpful to use the models in order to achieve a greater mechanistic understanding of what is observed. In particular, the prevalence of slower voltage depolarization in dendrites and disruption of spiking would benefit from some mechanistic insights that could be generalized to other model neurons. What factors (dendritic geometry, local input resistance etc.) lead to this behavior and does the presence of certain voltage-gated conductances enhance or disrupt these results? At it stands, the manuscript is largely descriptive of modeling data.

 The enhancement of IH in dendrites under in vivo-like inputs is noted but the mechanism (see above) is not explored. Why does IH conductance recruitment increase in dendrites? Are the results related to the slower time scale of voltage fluctuations under these conditions?

**Minor Issues:**

 Overall, the discussion focuses on IH. However, many of the results noted (e.g. slower voltage depolarization, enhanced difference between models under in vivo-like inputs) are very interesting and likely more generalizable than the specific issues of IH in OLM cells. It would be helpful to explore these issues in the context of what others have shown in terms of in vivo recordings and dendritic activity (e.g. SL Smith … M Hausser, 2013).

Is the work clearly and accurately presented and does it cite the current literature?

Yes

If applicable, is the statistical analysis and its interpretation appropriate?

Yes

Are all the source data underlying the results available to ensure full reproducibility?

Yes

Is the study design appropriate and is the work technically sound?

Yes

Are the conclusions drawn adequately supported by the results?

Yes

Are sufficient details of methods and analysis provided to allow replication by others?

Yes

Reviewer Expertise:

Slice and in vivo intracellular electrophysiology, biophysical modeling of neurons.

I confirm that I have read this submission and believe that I have an appropriate level of expertise to confirm that it is of an acceptable scientific standard, however I have significant reservations, as outlined above.

# Response

**Published:** 2020-06-06

**Authors:** Alexandre Guet-McCreight

**Affiliations:** University of Toronto, Canada

We thank the reviewers for their comments and detailed review of our manuscript. It has led to a better presentation and explanation of our work.

 We have revised our manuscript accordingly, changing and expanding two of the existing figures to exhibit results more clearly and providing more explanations. We hope that the changes made sufficiently addresses the concerns brought up by the reviewers. Please find below our specific responses (in **bold **font).

**Reviewer 1: Fernando Fernandez**

 In the manuscript by Guet-McCreight and Skinner, the authors use two variants of an OLM compartmental model to probe the impact of in vivo-like synaptic activity on membrane voltage fluctuation behavior and the differential recruitment of voltage-gated conductances.

 Overall the paper provides interesting insights into how synaptic inputs differentially impact voltage-gated conductances in dendrites and the cell body. Further, the impact of in vivo-like inputs on dendritic membrane voltage dynamics is particularly interesting as it dramatically increases the timescale of fluctuations and creates “plateau-like” depolarizations. In addition, the authors show that differences within the two biologically based OLM models can lead to substantial differences in dendritic voltage behavior and that these differences are enhanced when driven by in vivo-like inputs.

*Major Issues:*

 Although many of the results noted above are interesting. It would be helpful to use the models in order to achieve a greater mechanistic understanding of what is observed. In particular, the prevalence of slower voltage depolarization in dendrites and disruption of spiking would benefit from some mechanistic insights that could be generalized to other model neurons. What factors (dendritic geometry, local input resistance etc.) lead to this behavior and does the presence of certain voltage-gated conductances enhance or disrupt these results? At it stands, the manuscript is largely descriptive of modeling data.

**The reviewer is correct that passive property factors (geometry, resistance) contribute, and we have noted cell 1 and 2 differences (as shown in Fig 1).  That is, spikes propagate less in cell 2 relative to cell 1. We now also report the surface areas, input resistances and time constants for cell 1 and 2 as determined in Sekulic et al (2019) in the Methods.  We have also added dendritic diameter values for the locations shown.**

**We also present a new figure that zooms in on a 200 msec window for two dendritic locations (new Fig 3) which more clearly shows the contribution of the different channel types.  For example, one can now clearly see that there is an increased relative dominance of Ikdrf and IKCa in dendrites in vivo compared to in vitro and the disruption of firing can be seen with this along with the increased sodium currents as due to not being inactivated.  Additional wordings have been included in the paper.**

 The enhancement of IH in dendrites under in vivo-like inputs is noted but the mechanism (see above) is not explored. Why does IH conductance recruitment increase in dendrites? Are the results related to the slower time scale of voltage fluctuations under these conditions?

**This is brought up by reviewer 2 as well in the context that they do not find this result surprising, and the explanation that she provides is that “ *IPSCs are extremely effective in activating h-channels, as such, it is not surprising that h currents are more pronounced in the in vivo-like and sub-threshold conditions rather than during depolarizing current injections which would move the membrane outside the activation range for h-channels*”.**

**This is certainly a valid assessment and we have added this type of explanation to be more explicit in the results section discussing these findings. This can be seen more clearly in the edited and expanded Figures 3 and 4 where h-current accumulates in magnitude with increased durations of subthreshold voltage periods during the IVL states.**

*Minor Issues:*

 Overall, the discussion focuses on IH. However, many of the results noted (e.g. slower voltage depolarization, enhanced difference between models under in vivo-like inputs) are very interesting and likely more generalizable than the specific issues of IH in OLM cells. It would be helpful to explore these issues in the context of what others have shown in terms of in vivo recordings and dendritic activity (e.g. SL Smith … M Hausser, 2013).

**We agree that many of these aspects mentioned are generalizable across cell types and we have previously explored the potential impact of sensory-evoked stimuli during *in vivo*-like states in another hippocampal cell type (Guet-McCreight & Skinner 2019; Luo *et al*, 2020). Comparison to the results from Smith *et al*, 2013 in the context of mechanistic insight, is interesting, and our previous work suggests that during synaptic bombardment, sensory-evoked stimuli will largely be dampened (i.e. due to a reduced dendritic input resistance), which suggests the presence of more specialized mechanisms for sensory-evoked recruitment of cells (e.g. a rebalancing of synaptic bombardment, or plasticity mechanisms). We thank the reviewer for this suggestion and have added this reference as a point in our discussion.**
